# The Hematopoietic Cell Transplant Pharmacist: A Call to Action

**DOI:** 10.3390/pharmacy8010003

**Published:** 2020-01-02

**Authors:** Amber Clemmons

**Affiliations:** 1 College of Pharmacy, Department of Clinical and Administrative Pharmacy, University of Georgia, Augusta, GA 30912, USA; aclemmons@augusta.edu; 2 Department of Pharmacy, Augusta University Medical Center, Augusta, GA 30912, USA

**Keywords:** bone marrow, clinical pharmacy service, hematopoietic, responsibilities, roles, transplantation

## Abstract

Recently, the required training and credentials for as well as the various roles of the hematopoietic cell transplant (HCT) pharmacist have been endorsed by the leading organizations in cellular therapy, the American Society of Transplant and Cellular Therapy and the European Society of Blood and Bone Marrow Transplantation. While these documents establish the roles a HCT pharmacist can fulfill within the multi-disciplinary team, few reports have evaluated the impact of the HCT pharmacist on clinical, financial, or quality outcomes. Further, a paucity of information has been reported on types of practice models, such as the use of collaborative practice agreements, or described effective methods to overcome the barriers to the increased utilization of HCT pharmacists. Herein, a brief summary of available information is provided to aid readers in understanding the state of the science for pharmacists practicing in this specialty with the goal to stimulate further research to justify the roles of HCT pharmacists and the correlation of such research to various outcome measures. Practitioners are encouraged to build upon this existing knowledge to create the novel integration and elevation of pharmacy practice to improve outcomes for patients, providers, and payors.

## 1. Introduction

The roles of the clinical pharmacist, including those specifically practicing within the hematology/oncology setting, have been well delineated and justified [1,2,3]. Given the specialty knowledge required, the overall complexity of patient care often spanning a prolonged inpatient stay with subsequent numerous outpatient visits over an extended period of time, potential impending provider shortage, and the existing multi-disciplinary team approach to patients undergoing hematopoietic cell transplantation (HCT), the roles and value of a pharmacist within this sub-specialty setting were only recently established in both the Unites States and Europe [4,5]. Herein, the progress in defining responsibilities of the HCT pharmacist, with a succinct summary of evidence supporting such roles and the affiliated impact on outcomes, as well as barriers and future needs, are briefly reviewed.

## 2. Discussion

Per a survey of transplant center directors administered in 2012, pharmacists are incorporated into the HCT team in over 85% of centers, regardless of transplant center size or age of patient population [6]. With an emphasis on value-based care [7] and the anticipated shortage of providers to adequately cover the growing rate of HCT world-wide, a need exists to establish the exact roles of the HCT pharmacist and affiliated improvement in outcomes.

Much progress has been made in the past decade regarding the advancement of the HCT pharmacist. In 2009, given the rising utilization of HCT, the National Marrow Donor Program initiated a three-year System Capacity Initiative (NMDP SCI) to address the impending capacity barriers in this specialty area [8]. Regarding pharmacy practice, the workgroup recommended the following: establishing minimum standards for pharmacists and endorsement by Foundation for the Accreditation of Cellular Therapy–Joint Accreditation Committee ISCT EBMT (FACT–JACIE), creating formal HCT training opportunities for pharmacists, and encouraging use of collaborative practice agreements (CPAs) [9,10]. Achievement of these outcomes marks the initial progress within HCT pharmacy practice. While previous editions only required pharmacists to be involved in the chemotherapy process (i.e., preparation, verification, and dispensing, compared to protocol or standardized regimen), as of 2015 the sixth edition of FACT-JACIE recognized pharmacists as core members of the transplant team who will also “be involved in…the pharmaceutical management of cellular therapy patients” [11]. The revised standard highlights the recognition of pharmacists as key team members who, like other HCT practitioners such as advance practice providers (APPs), now require ten hours of relevant continuing education annually. The FACT-JACIE standard delineates required topic areas for training and education of the HCT pharmacist (e.g., cellular therapy process, therapeutic drug monitoring, dose adjustment for organ function, drug interactions, etc.) [12]. Addressing this need, the American Society of Transplant and Cellular Therapy (ASTCT, formerly ASBMT) Pharmacy Special Interest Group (SIG) has provided the “Fundamentals of HCT Training Course” and the “Beyond Fundamentals” course [13]. Lastly, addressing the NMDP SCI’s recommendation to encourage CPAs to allow pharmacists to function as extenders and relieve some of the medication specific workload off of physicians and APPs the ASTCT Pharmacy SIG published a survey on the use of CPAs in HCT [14], which is detailed in the section below.

The initial characterization of the roles performed by the HCT pharmacist was recorded by the NMDP SCI from a survey presented in 2011 [15]. Based on the results of the survey, the workgroup concluded a need existed to define the roles of HCT pharmacist and standardize the delivery of care. Only recently has a consensus on the precise responsibilities and activities as well as description of competencies of the HCT pharmacist been established. First, the ASTCT Pharmacy SIG created the “Roles of the HCT Pharmacist” statement, which was subsequently endorsed by other organizations, including the Hematology/Oncology Pharmacy Association (HOPA) [16]. This document states “The HCT clinical pharmacist is an integral member of the multidisciplinary HCT team who provides a variety of pharmacy and educational services … to optimize collaborative, patient-centered care focused on patient safety.” The document succinctly lists the main activities falling under categories of medication/disease state management, transitions of care, education, and research/quality improvement (Table 1). Expanding upon this document, a review article sponsored by the ASTCT Pharmacy SIG goes further to justify, via supporting evidence, the need of pharmacist oversight and/or participation in each activity area [4]. This article aimed to provide a summary of data supporting the HCT pharmacist in each role to be utilized by those justifying positions or expanding service models, as well as to stimulate current practitioners to continue to research the impact of the HCT pharmacist. As this article focused on practice within the United States, recently the European Society of Blood and Bone Marrow Transplantation (EBMT) created a Pharmacist Committee that devised their own consensus recommendations on the competencies and roles of the clinical pharmacist and clinical pharmacologist in HCT [5]. While the recommendations align well with the aforementioned ASTCT recommendations, the incorporation of the clinical pharmacologist, whose training and role may vary among countries, and commentary on lack of formal education and certification programs in European countries is unique to the EBMT statement. Both recommendation statements endorse similar roles and responsibilities in which an HCT pharmacist should participate; however, the documents are structured differently. While the ASTCT recommendations list general activities for the HCT pharmacist under various domains (see Table 1 below), the EBMT recommendations provide a checklist of required and optional activities for the clinical pharmacist and clinical pharmacologist. Both documents endorse pharmacist roles with medication management, patient care, transitions of care, education, and research. Notably, differences between the two documents can be seen in specific areas, such as participation with financial assistance and trainee education being listed as optional activities in EBMT document. Additionally, the ASTCT document includes commentary on advocacy efforts that is not included in EBMT checklist, likely reflecting differences in overall medical and payment models between the United States and European countries. Lastly, the EBMT document includes pharmacist role with managing advanced therapy medicinal products, such as chimeric antigen receptor T-cells (CAR-T), which are not specifically mentioned in the ASTCT document; however, this is likely due to the ASTCT publication occurring concurrent with the initial approval of those products in the United States. Overall, both documents are endorsed by the leading HCT organizations highlighting the clear acceptance of the integral role of the pharmacist/pharmacologist in HCT practice.

### 2.1. Supporting Evidence for the HCT Pharmacist

As mentioned, data supporting these roles have been reviewed in the ASTCT Pharmacy SIG’s article, to which readers are referred for full details [4]. Since this was not a formal review of the literature, a subsequent systematic review of pharmacists’ contributions was conducted by Barboza-Zanetti and colleagues. This review included seven articles published by April 2017: four studies assessed outpatient clinic, two inpatient, and two a mixture of settings; four studies assessed autologous and allogeneic HCT populations; and three assessed only allogeneic HCT population. HCT pharmacist activities reported in these studies included medication management, discussion with clinical teams, medication reconciliation, adherence, education of teams and patients, and participation in guideline development. Overall, all studies indicted the usefulness of these HCT pharmacist services. Activities were correlated with improved patient clinical and nutritional status, maintenance of immunosuppressive levels, prevention and control of pharmacotherapy-related problems, facilitation of adherence, and economic and humanistic (time savings, satisfaction) advantages [17]. Notably, correlating pharmacist activities to specific outcomes is challenging due to the multi-disciplinary nature of HCT; therefore, primary endpoints of relapse, survival, etc., are difficult to target, which has led to the selection of secondary outcomes (immunosuppression levels, etc.) instead within these studies [18]. Reported rates of acceptance of pharmacist recommendations in these reports indicate the general support of pharmacist roles by providers [17].

Few other reports have been disseminated since the systematic review. Morrison and colleagues conducted a review of the literature specific to adherence in the HCT population given the general correlation of nonadherence to poor outcomes [19]. This review included five studies that assessed inpatient and/or outpatient populations and utilized a variety of methods to assess adherence, mainly patient report. Germane to our focus here, only one report included adherence promoting intervention, which was pharmacist intervention of discharge consultation with six scheduled follow-ups to review for drug-related problems. Self-reported adherence was improved over the course of follow-up for those who continued to see the pharmacist, although no comparison was made to adherence rates for patients not seen by the pharmacist. Of note, this study was included in the systematic review by Barboza-Zanetti and colleagues above. Since medication education to patients and caregivers and review of adherence is central to all pharmacists, this presents a clear area for further research to correlate pharmacist activities with outcomes related to adherence.

Lastly, Cao and colleagues recently presented their implementation and evaluation of specific outpatient pharmacy services showing a systematic approach to identify and resolve medication access and provision of education leading to cost-savings and zero delays in medication access [20]. The importance of integration of pharmacy services early in the HCT process to educate patients and ensure access to medication was confirmed. This report also showed the value of incorporation of pharmacy technicians, prescription assistance teams, and pharmacy students. Further reports detailing the benefit of specific pharmacy services, such as education and medication access, on financial outcomes can aid other practices in justifying positions to provide these activities.

HCT pharmacists are encouraged to review the aforementioned existing literature and use this to stimulate evaluations in their current practices to create further reports on the impact of the HCT pharmacist. Additional formal data supporting the impact on clinical, financial, and humanistic outcomes can aid in the expansion of the HCT pharmacist across many programs.

### 2.2. Formal Recognition of Delegated Activities for the HCT Pharmacist

Utilizing all members of the multi-disciplinary team, including the pharmacist, to the fullest extent of their knowledge, skill, and abilities may alleviate part of the workforce shortage in HCT [7]. As mentioned earlier, CPAs have been promoted to formalize the delegated roles of the HCT pharmacist. CPAs are official contracts between physician(s) and pharmacist(s) to allow the later to manage patients’ medication therapy, as delineated in the agreement. Given the complexity of medication regimens for HCT patients, number of medications with therapeutic drug monitoring, and potential for relevant drug-drug interactions, the incorporation of pharmacist-led medication management via CPAs has been recommend as part of value-based care initiatives [7]. Further, the alignment of pharmacist roles with quality metrics and has been stated [21]. While a paucity of data exists regarding benefits of CPAs specifically in HCT relating to outcomes, metrics, and cost-savings, correlation with improvement in these outcomes has been shown in medicine and oncology settings [18]. In a recent survey of HCT pharmacists, 46% of respondents had an existing CPA while an addition 21% stated they were planning to implement CPA. Activities covered under CPAs varied among sites but often included changing or discontinuing medications, therapeutic drug monitoring, or dose modifications (e.g., renal dose adjustment), as well as managing supportive care, including pain management, and comorbid conditions/chronic disease states [14]. Given the increased utilization of CPAs but notable variation in delegated activities, further reports of effective CPA models and correlation with outcomes are needed to aid in incorporation of CPAs into more HCT centers.

Notably, CPAs are only one method to accomplish the mission of allowing pharmacists to practice at the ‘top of their license’. CPAs can be limited by inability to obtain reimbursement for pharmacist services, lack of willing provider collaborator, lack of back-up coverage, individual state scope of practice rulings, burdensome regulation, etc. [14,22]. Therefore, other options have been explored and encouraged. Privileging is the “process by which a healthcare organization, having reviewed an individual healthcare provider’s credentials and performance and found them satisfactory, authorizes that individual to perform a specific activity”. Privileging within an organization can allow for maximum participation of pharmacists in the pharmacists’ patient care process to improve access to care and clinical outcomes while reducing costs [23]. Like CPAs, privileging has barriers to implementation and potential problems, such as need for administrative oversight, ongoing review of pharmacist practitioners, and need for buy-in from providers [23]. It has been noted that state laws can often impede implementation of CPAs and/or privileging [22]; therefore, advocacy efforts are warranted to resolve these barriers. Further, the ongoing efforts related to pharmacist provider status can impact the operationalization of certain pharmacist roles in the future. Assessment and dissemination of both CPA and privileging models in the HCT setting is necessary to demonstrate the value and advocate for further integration of pharmacists as nonphysician providers. HCT centers with established CPA or privileging models for HCT pharmacist are highly encouraged to disseminate their credential and training requirements, ongoing evaluation process, methods taken to overcome barriers to implementation, and importantly any affiliated outcomes post-implementation to aid other centers. Institutions may consider presenting their models at pharmacy conferences in “best practice” sessions or through publications in journals. Outcomes assessed pre- and post- implementation of any new CPA or privileging can vary. However, inclusion of clinical outcome metrics, such as percentage of patients within goal immunosuppression level range, for example, and humanistic metrics, such as time saved by physicians, can be reported to justify pharmacist services.

### 2.3. Considerations in HCT Pharmacy Practice

While existing data support the vital role of HCT pharmacist, expanding practice models, and impact on outcomes, practitioners and administrators should be knowledgeable on the critical factors for success of these pharmacists. Notably, the HCT pharmacist may be at high risk of burnout and moral distress. In a 2015 survey, more than half of HCT pharmacists had scores revealing burnout, which was higher than the overall rate of 40% for all HCT practitioners surveyed [24]. Further, HCT pharmacists had the highest scores of moral distress compared to other HCT practitioners surveyed. Survey respondents with burnout were more likely to report both poor work-life balance and career satisfaction. However, overall rate of career satisfaction of HCT pharmacists was high, with >80% respondents stating they agreed or strongly agreed with statement “I am satisfied with my career in HCT.” Authors of the survey postulated reasons for the high rates of burnout and moral distress as well as affiliated poor work-life balance: required time spent in additional distribution or non-HCT coverage responsibilities, ambiguity or conflict in roles between the pharmacist and other team members, and small centers with no qualified back-up for time off [24].

Given the growing recognition for the importance of workplace mental health and potential relationship of burnout with poor job performance or loss of employees, these items present clear areas for assessment and intervention. Before implementing HCT pharmacist roles, CPAs, or privileging, each institution should consider how to ameliorate these potential complications. Ensuring the designated responsibilities are feasible, supported by local infrastructure, and ensure optimal workflow is important when designing or expanding an HCT pharmacist position.

## 3. Conclusions

Practitioners and administrators in the United States and Europe now have endorsed statements defining the role and stating the value of the HCT pharmacist. A clear need exists for further study relating specific pharmacist roles to improvement in various quality metric, clinical, humanistic, and financial outcomes. Further, institutions are encouraged to evaluate and report how to optimally structure practice models, including CPAs and privileging. Given the heterogeneity in HCT programs, no ‘one size fits all’ approach is recommended [6]. Rather, increased reporting of various centers’ models and experiences would benefit other programs’ revision or expansion efforts. Alignment of individual program infrastructure, financial, and personnel resources with reported best practices is advised. Pharmacists in the HCT setting are therefore strongly encouraged to formally evaluate and report on their daily activities and practice models with a correlation to outcomes to further justify and enhance the care of the HCT patient.

## Figures and Tables

**Table 1:** Summary of the activities of an hematopoietic cell transplant (HCT) pharmacist [4,16].

Domain	Activities
Medication management and monitoring	✓ Provide thorough medication profile review ✓ Participate in interdisciplinary rounds ✓ Manage chemotherapy processes ✓ Assist with therapeutic drug monitoring ✓ Provide medication therapy management ✓ Manage anti-infective therapies and promote stewardship
Patient care	✓ Assist in symptom management ✓ Optimize graft-versus-host disease management ✓ Facilitate post-transplant vaccination
Transitions of care	✓ Assist with transitions of care, including in hospice/palliative care when applicable ✓ Facilitate access to medications available through patient assistance programs
Education	✓ Educate patients, caregivers, as well as healthcare providers and trainees
Research and quality improvement	✓ Contribute to institutional and collaborative research and scholarly activities ✓ Assist with policy and guideline development ✓ Serve as a patient and professional advocate ✓ Monitor, evaluate, and report transplant-related outcomes to assist in improvements in clinical practice
